# Inducing resistance to the misinformation effect by means of reinforced self-affirmation: The importance of positive feedback

**DOI:** 10.1371/journal.pone.0210987

**Published:** 2019-01-22

**Authors:** Malwina Szpitalak, Romuald Polczyk

**Affiliations:** Institute of Psychology, Jagiellonian University, Kraków, Poland; Ghent University, BELGIUM

## Abstract

The misinformation effect is one of the major threats for the quality of witness testimony. It involves including of information that is inconsistent with the course of an event, and which originates from sources other than the event itself, into a witness's report of the event. In the present article research is presented aiming at reducing the tendency to rely on misinformation. After viewing a video clip, participants received a post-event narrative describing the events in the film which in the misled group included some incorrect information about the clip. They were then administered reinforced self-affirmation (RSA), a technique aiming at boosting self-confidence in order to increase the tendency to rely on own memory instead of external cues. This technique consists of self-affirmation by means of writing down one’s greatest achievements in life and manipulated positive feedback. Feedback about memory, perception and independence of judgements was analyzed. All types of feedback effectively reduced the misinformation effect. Mediation analyzes confirmed that RSA operates *via* increased self-confidence or self-independence.

## Introduction

The aim of the article is to present the technique of reinforced self-affirmation, in various variants, as a method of immunization against misinformation in the context of witness testimony. Developing methods to improve the quality of human testimony is important as it provides important evidence in court [1,2,3,4]. However, witness testimonies are often unreliable and inaccurate (e.g. [5,6,7]), which is due to the natural conditions in which human memory operates [8,9]. Moreover, a witness is often confident in the accuracy of his or her objectively incorrect testimony. Meta-analyses show that the correlation between the witness's confidence and the accuracy of his or her testimony is either statistically insignificant [10] or significant, but weak [11,12]. However, a confident witness seems to be more credible to the court [13]. Obviously, erroneous testimony can have significant social consequences.

The mechanisms by which errors enter witness testimony are varied. One of them is the misinformation effect. It is defined as the inclusion of information that is inconsistent with the course of an event, and which originates from sources other than the event itself, into a witness's report of the event [14]. This effect is sometimes described as one of the most important discoveries of psychology [15], and its significant impact on memory functioning is also pointed out [16].

The most frequently used procedure to investigate the effect of misinformation consists of three stages [6,17]. First, the original material is presented(the equivalent of the event the witness later testifies about). Then, after some time, the subjects are presented with the postevent material, which in the experimental group contains information that is incongruent with the original event. The last step is the test of the memory of the original material.

There are two broad types of reasons why people give in to misinformation. The first one includes some malfunction of the memory process. Theories based on this assumption include, among others, parallel traces theory [18], the CHARM model [19], fuzzy trace theory [20], activation-based theory [21], retrieval-induced forgetting [22], memory reconsolidation [23], and the source misattribution theory [24,25,7]. The second class of theories do not assume that memory malfunction is necessary for the misinformation effect to occur [26] and point out that it is possible that a witness testifies in accordance with the misinformation in spite of the fact that they remember all information necessary to provide correct testimony [5,27,28,29]. For example, if a yellow car was visible in the video, subsequently described as red, some participants may think that they saw a yellow car, but they also believe the car was red in the description of the story. When asked about the colour of the car in the video, such participants experience obvious subjective discrepancies which they have to resolve. Some participants choose to ignore the postevent material and to rely on their own memories. As a consequence, they give the correct answer—the car was yellow. Some other however misbelieve their memories, assume that the description must have been correct, and give a wrong answer based on it—that the car was red.

In the light of existing research the main reason for yielding to misinformation by people aware of the discrepancies between the original material and the misinformation is doubt in their own memory [5]. This article presents research on a technique of immunizing against misinformation which aim at increasing the confidence of a witness as regards some particular psychological properties reinforced self-affirmation (RSA).

RSA combines two techniques: self-affirmation and positive feedback. It was designed for the kind of eyewitnesses mentioned above—those who remember correctly the original event yet tend to report information consistent with the erroneous postevent material rather. It is based on the assumption mentioned above—that one of the main reasons why a witness yields to misinformation is doubting the accuracy of their own memoirs [5,30]. It may be assumed that in the case of people who have a correct memory of the original material the increase in confidence in the quality of their own memory should result in a decrease in the tendency to rely on external sources of information.

This assumption is not new. Attempts to strengthen self-confidence or self-esteem with regard to the functioning of the witness's memory by giving them positive feedback were made earlier. For example, Tata and Gudjonsson [31] used positive feedback manipulation in the context of Gudjonsson's and Clark's [32] [33] interrogative suggestibility procedure. The study found that positive feedback made people less susceptible to suggestions compared with negative feedback.

The above-mentioned result supports the thesis that an effective technique of immunizing against misinformation should enhance self-confidence, in particular confidence in the accuracy of one's own memories. Positive feedback should be a component of such a technique, providing the individual with information about the good quality of the performance on a task. It has been shown that an individual's awareness that they have succeeded or failed in their task has a significant impact on their behaviour [34], affect [35] and confidence [36,37]. Also, positive feedback about the quality of the current task increases motivation [38] and self-confidence, including confidence about memory performance [39,40]. In the Szpitalak [41] study, however, positive feedback alone did not have any impact on susceptibility to misinformation in the three-stage paradigm described above. Therefore, Szpitalak [41] added a second component: the act of self-affirmation. It was expected that the individual, focused on the positive self-characteristics generated internally (by means of self-affirmation), would be more willing to accept positive feedback as a kind of self-affirmation confirmed externally.

The self-affirmation activity consisted in the respondents writing down their greatest achievements in life. It has been suggested that concentrating on one's achievements is an effective method of self-affirmation [42], and the subject's belief that he or she has been successful in the past increases confidence, just as recollections of experienced failures decreases confidence [43]. The act of self-affirmation should therefore contribute significantly to the self-confidence of the subject. It also seems that the subject's involvement in the act of self-affirmation fosters a state of self-concentration. This in turn may enhance the efficacy of the feedback, because the individual focused on the self seems to be more willing to learn about him or her from the environment [44] and to analyze it more carefully [45].

In sum, it has been empirically demonstrated that both positive feedback [36] and self-affirmation [46] increase self-confidence. Self-confidence, in turn, is a positive predictor of the level of performance on a task [34]. In the area of forensic psychology, the relationship between a witness's self-confidence and the quality of his or her testimony was also demonstrated: self-confidence has been found to be negatively linked to susceptibility to suggestions [47] and to memory conformity [48]. It was therefore expected that RSA, combining positive feedback and self-affirmation would boost self-confidence and therefore promote relying on one's own memory and not on external sources, thus endorsing resisting misinformation.

According to existing research, RSA is effective in reducing the misinformation effect [41,49,50] and susceptibility to suggestions in the paradigm of interrogative suggestibility [51]. However, not every kind of positive feedback proved effective. So far, it was demonstrated that RSA was effective when it included positive feedback concerning memory quality (that is, the area related to the task at hand—the misinformation effect procedure). Positive feedback concerning attention or general ‘morality’ was not effective [52]. In the present paper, the relevance of positive feedback is further explored. It is also analyzed whether RSA really involves increasing the self-confidence of a person in a specific area.

### Aims and hypotheses

The main aim of the present study was to examine whether the positive feedback in the RSA procedure is effective only when it concerns memory, or whether it may concern other areas, without losing its effectiveness. The first study explored RSA with feedback concerning memory (MemRSA) and RSA with feedback about perception abilities (PerRSA). Both were expected to effectively reduce susceptibility to misinformation, according to the rationale presented above.

In a similar vein, boosting confidence about perception ability was expected to promote giving correct answers consistent with one’s own accurate memory. It is possible that when confronting discrepancies with what seems to be one’s own memory and the content of the postevent material, some participants may be unsure whether they indeed saw a given detail in the original video clip. Increasing confidence about perception abilities should reduce such doubts and promote correct answers based on one’s own accurate memory.

Apart from boosting confidence about memory and perception quality, it was also expected that activating convictions that one is very independent in their judgements (IndRSA) would reduce yielding to misinformation. This hypothesis is based on similar reasoning as in the case of MemRSA and PerRSA and on the same basic premise that there are participants who at the moment of the final memory test remember both the original and postevent materials. Making such participants believe that they are independent in their judgements should increase the tendency to rely on their own memory and therefore decrease the misinformation effect.

In sum, it was hypothesized that: MemRSA increases confidence about memory which in turn reduces the tendency to ignore one’s own correct memory; PerRSA increases confidence about perception ability which also in turn reduces to tendency to rely on sources of information other than one’s own memory; and IndRSA evokes beliefs that one is independent in their judgements and this also makes the participants to rely on their own memories. Such hypotheses are mediational in their nature, therefore analyses of mediation were planned. In order to perform them, measures of confidence in memory and perception ability as well as a measure of perceived independence in judgements were included in the procedure.

## Experiment 1

In Experiment 1, MemRSA and PerRSA were explored. The main basic hypothesis concerned the replication of the misinformation effect. If there were no reliable misinformation effect, any technique aiming at reducing it would be pointless. Therefore, it was expected that the misinformation effect would be replicated (Hypothesis 1): the mean number of answers consistent with misinformation should be higher in the misled group than in the control one in which no misinformation was present. Hypothesis 2 stated that MemRSA would be effective: in the misled group, the mean number of answers consistent with misinformation should be lower in the MemRSA group than in the control groups in which RSA was not applied (NoRSA). Similar hypothesis was formulated in the case of PerRSA: in the group in which it was performed, the mean number of answers consistent with misinformation should be lower than in the NoRSA group (Hypothesis 3). We also compared the efficacy of MemRSA and PerRSA even though we did not have a priori hypotheses about the outcome of this comparison. As the hypotheses were formulated *a priori* (in the technical sense, not as a preregistration), in line with existing recommendations [53,54,55], no overall ANOVA was performed and instead focused comparisons in the form of planned contrast were calculated. Finally, it was expected that memory confidence would mediate the relationship between MemRSA and yielding to misinformation (Hypothesis 4), as well as confidence about perception ability would mediate the impact of PerRSA on yielding to misinformation (Hypothesis 5).

### Methods

#### Participants

Two hundred and twenty-six participants, students of various schools (138 women, 88 men) aged between 15 and 18 took part in the study (M = 16.6; SD = 0.7). The experiment was performed during normal classes. The number of participants was determined *a priori* in order to have a reasonable chance of detecting weak small effects. Participation was anonymous, and no reward was given for it. A verbal consent was obtained, as the participation was announced as fully anonymous, thus making it impossible for the participants to sign a written consent. Ethical approval for this experiment and Experiment 2 was obtained from “Komisja Etyki Instytutu Psychologii Uniwersytetu Jagiellońskiego” [Ethical Committee, Institute of Psychology, Jagiellonian University].

#### Materials

A four-minute video clip showing burglary and theft was used as the original material. A short description of the content of the film was used as the postevent material, which in the misled group contained six details changed in relation to the content of the film or added to it. The memory test of the original material consisted of 12 open-ended questions, six of which referred to the misled details. For example, no gold watches were stolen by the thieves, the postevent material suggested that they did so, and the question on the final memory test asked what did the thieves steel (the content of the postevent material and the questions included in the memory test are given in Supporting Information, S4 and S5 Files).

The RSA procedure consisted of two parts. First, the participants in the experimental groups had to write down their greatest life achievements. The participants in the control condition wrote down their route from home to school. Afterwards, the participants from the MemRSA condition were memorizing as many unrelated nouns as possible from a list of 60. After two minutes the list was removed and answer sheets were distributed. They had numbered slots in which the participants were to enter the nouns so that they were able to see how many items they were able to remember. They were then given manipulated positive feedback consisting of written information about the average number of nouns seemingly ‘usually remembered’, which in fact was 1.5 *SD* lower than the real number, therefore most subjects scored above ‘the average’. In the control condition, no feedback was given. This task took about six minutes. In the PerRSA group, instead of memorizing nouns the participants were asked to mark differences between two abstract images shown to them on a horizontal sheet of paper. Afterwards they were to count and write on the sheet the number of differences found and received feedback on their performance which consisted in a mean number of differences ‘usually found’ which in fact was 1.5 *SD* lower than the real mean established in a pilot study.

#### Procedure

The research took place during classes in groups of several people. The participants were informed that the experiment concerned ‘psychological conditions of processing visual material’. Afterwards, the participants were shown a video clip without sound and completed some filler questionnaires for 15 minutes. After that they were asked to read a short description of the film, seemingly ‘in order to answer some questions about the person who wrote the description’. Next, the RSA procedure was applied. Afterwards, a short questionnaire in the form of a 100mm visual analogue scale (VAS) was administered. Two of its questions served as the manipulation check for the RSA manipulations; the first one referred to the confidence about memory: ‘Rate by marking in a vertical line below how confident you feel at this point that you have a good memory of the content of the film’, the second one—about perception: ‘Rate by marking in a vertical line below how confident you feel at this point that you have noticed much of the content of the video’. Finally, the memory test about the original video clip was administered after which the participants were fully debriefed. At the end of the experiment, the participants were debriefed. In sum, the study was based on a 2 × 3 experimental design: misinformation (present / absent) × RSA (MemRSA / PerRSA / NoRSA).

### Results and discussion

#### Manipulation checks

As expected, the NoRSA group (*M* = 57.56, *SD* = 23.11) had statistically significantly lower memory confidence compared with the MemRSA group (M = 69.81, *SD* = 20.85, difference between means (*D*) = 12.25, *F*(1, 223) = 12.02, *p* < .001, ƞ^2^ = .05) as well as compared with the PerRSA group (*M* = 66.03, *SD* = 22.91, *D* = 8.47, *F*(1, 223) = 4.96, *p* = .027, ƞ^2^ = .02). This confirms the efficacy of MemRSA in boosting confidence about memory, but it should be acknowledged that also PerRSA increased this confidence.

The effectiveness of RSA concerning perception was also confirmed: The NoRSA group (*M* = 60.00, *SD* = 22.66) had statistically significantly lower self-confidence about perception compared with the PerRSA group (*M* = 67.80, *SD* = 22.07, *D* = 7.80, *F*(1, 223) = 4.03, *p* = .046, ƞ^2^ = .02), as well as with the MemRSA group (*M* = 68.04, *SD* = 23.09, *D* = 8.04, *F*(1, 223) = 4.96, *p* = .027, ƞ^2^ = .02). The measures of memory and perception confidence were not correlated (*r* = .09, *ns*). In sum, the analyses confirmed the effectiveness of the RSA, both the one related to feedback on memory and perception.

#### Main analyses

Descriptive results concerning the mean number of answers consistent with misinformation are given in Table 1. The range of the means is from 0 to 6.

**Table 1:** Means (*SD*s) of number of answers consistent with misinformation across experimental conditions in Experiment 1 (range: 0–6).

Feedback in RSA	No misinformation	Misinformation	Total
No feedback	0.09 (0.29)	3.17 (2.01)	1.67 (2.12)
Memory	0.35 (0.69)	1.88 (1.49)	1.04 (1.36)
Perception	0.25 (0.44)	1.84 (1.73)	1.17 (1.55)
Total	0.24 (0.54)	2.27 (1.83)	0.24 (0.54)

As predicted in Hypothesis 1, the misinformation effect was replicated: in general, the number of answers consistent with misinformation was higher in the misled than in the control group by about two points on a scale from 0 to six (*D*) = 2.03, *F*(1, 220) = 138.85, *p* < .001, ƞ^2^ = .39). The second hypothesis was also confirmed by means of a planned simple contrast comparing the mean yielding to misinformation in the MemRSA and the NoRSA group among the misled participants: *D* = -1.29, F(1, 220) = 18.84, *p* < .001, ƞ^2^ = .08.

In order to verify the third hypothesis, the results of the PerRSA and the NoRSA group were compared, and it turned out that memory was significantly more accurate in the PerRSA than in the NoRSA group (*D* = -1.33, *F*(1, 220) = 19.21, *p* < .001, ƞ^2^ = .08), confirming this hypothesis. Moreover, the effectiveness of both RSA procedures seemed not to differ (*D* = -0.04, *F*(1, 220) = 0.02, *p* = .902, ƞ^2^ < .01). The differences between RSA groups were not significant among the non-misled participants (NoRSA *vs*. MemRSA: *D* = 0.26, *F*(1, 220) = 0.80, *p* = .373, ƞ^2^ < .01; NoRSA *vs*. PerRSA: *D* = 0.16, *F*(1, 220) = 0.24, *p* = .626, ƞ^2^ < .01; MemRSA *vs*. PerRSA: *D* = -0.10, *F*(1, 220) = 0.10, *p* = .753, ƞ^2^ < .01).

To verify the mediational models postulated above, mediation analyses were performed, by means of the software PROCESS v. 3.0 [56]. Two analyses were performed, the first one with memory confidence as the mediator, the second one—with confidence about perception ability. The number of answers consistent with misinformation was the dependent variable. RSA served as the predictor. As it was a nominal variable (three groups: NoRSA, MemRSA and PerRSA), it was set as a multicategorical predictor in the PROCESS, and indicator contrasts were used to analyze the differences between groups. The NoRSA served as the reference group, and the MemRSA and PerRSA groups were compared to it. Confidence intervals for the indirect effects generated by the bootstrap method were used to determine whether the indirect effects were significant. 95% confidence intervals were calculated, which correspond to the alpha level of 0.05. Table 2 presents the results of the mediational analyses.

**Table 2:** Results of mediation analysis—Experiment 1.

Contrasts for the predictor	Mediator	*b* [95% CIs]
NoRSA—MemRSA	Memory confidence	-0.28 [-0.60, -0.03]
	Perception confidence	-0.07 [-0.27, 0.06]
NoRSA—PerRSA	Memory confidence	-0.15 [-0.39, 0.06]
	Perception confidence	-0.12 [-0.37, 0.08]

Predictor: RSA; Dependent variable: Number of answers consistent with misinformation

*b*: regression coefficient for the indie

The mediation: MemRSA > Memory confidence > Yielding to misinformation proved significant, as the 95% CIs for the indirect effect did not contain zero. This confirms the Hypothesis 4: the procedure for boosting memory confidence effectively reduced the misinformation effect, and this effect was mediated by memory confidence but not by perception confidence. However, a similar Hypothesis 5 concerning the confidence about perception was not confirmed, as the confidence intervals for the indirect effects included zero.

In sum, the results of Experiment 1 confirmed almost all the hypotheses. The misinformation effect was replicated with quite a substantial effect size. Increasing confidence relating to memory and perception resulted in reducing the vulnerability to give answers based on an external source of information. Apparently, subjects convinced that their memory and perception ability is of good quality decided to rely on their own recollections, instead of rejecting them and choosing to give answers based on other sources. Additional mediational hypotheses were confirmed in the case of memory confidence but not perception confidence: it was found that that MemRSA indeed influences yielding to misinformation *via* increased memory confidence.

## Experiment 2

In Experiment 2, MemRSA and IndRSA were examined. As in Experiment 1, a replication of the misinformation effect was expected (Hypothesis 1). Hypotheses 2 and 3 stated that MemRSA and IndRSA would reduce the misinformation effect: it was expected that in the misled group, the mean number of answers consistent with misinformation would be smaller in the MemRSA and IndRSA groups than in the NoRSA group. In addition, it was expected that combining both feedbacks: about memory and independence of judgements (MemIndRSA) would be more effective than MemRSA alone (Hypothesis 4a), as well as IndRSA alone (Hypothesis 4b).

Mediation analyses were performed to verify the hypothesis that the impact of MemRSA on yielding to misinformation is mediated by memory confidence (Hypothesis 5), as well as that the impact of IndRSA is mediated by subjectively perceived independence of judgements (Hypothesis 6). As in Experiment 1, planned contrasts were calculated to analyze the hypotheses concerning differences between groups, and the software PROCESS [56] was used to analyze the mediation models.

### Method

#### Participants

Four hundred and thirty-six participants, students of various universities as well as other persons recruited *via* advertisements in newspapers and in the Internet (304 women and 132 men) took part in the experiment. Their mean age was 23.0 (*SD* = 6.8, range 15–65 years). The experiment was run in groups of several participants. The sample size was determined by funds available as the participants were given a reward of about 7 €. Written signed consent was obtained from all participants.

#### Materials and procedure

The same materials as in Experiment 1 were used for the misinformation procedure and the MemRSA. A procedure for inducing subjectively perceived independence of judgements was applied which has already been used in previous research [50,52], administered after the standard part of RSA consisting in writing down one’s greatest achievements in life. It consisted of a computerized personality test presented on a computer screen, on which questions concerning self-efficacy, self-esteem and need for closure were presented, taken from various questionnaires. After the test, the computer presented ‘the results’, which were as follows: ‘You are assertive and independent in thinking. You love to present your own opinions, although sometimes you feel uncertain for no apparent reason. You cope well with problems in your life, but sometimes you feel upset in social interactions. You don’t like crowded places. You believe in having own opinions, not relying on others. You believe your intuition, even if you don’t realize it’. After this, all participants did the memory task as in Experiment one. Depending on the types of feedback, following experimental groups were created: MemRSA, in which positive feedback about memory but not about independence of judgements was given; IndRSA, in which no feedback about memory but one about the independence was presented, and MemIndRSA, in which both types of feedback were administered.

Memory confidence was measured by means of a 100 mm VAS scale, as in Experiment 1. In addition, a short questionnaire with five questions, e.g. “I believe that my memory of how well I remember the content I learned can be judged:”. Answers were given on a 7-point Likert-like scale, from 1: Very low, to 7: Very high, making a range from 5 to 35 points. Similar methods were applied to measure subjective independence of judgements: a 100 mm VAS scale, and a questionnaire with five questions, e.g. “I make decisions on matters that are important to me completely on my own”. The scoring was the same as in the case of memory confidence, with the range of answers from 5 to 35. At the end, the participants were debriefed.

The study was based on a 2 × 4 experimental design: misinformation (present / absent) × RSA (MemRSA / IndRSA / MemIndRSA / NoRSA).

### Results and discussion

#### Manipulation check

As expected, the level of memory confidence as measured by the questionnaire was significantly lower in the NoRSA group (*M* = 21.45, SD = 4.15) compared to the MemRSA group (*M* = 23.53, *SD* = 5.90, *D* = 2.08, *F*(1, 432) = 9.66, *p* = .002, ƞ^2^ = .02) and to the MemIndRSA group (*M* = 23.41, SD = 5.65, *D* = 1.96, *F*(1, 432) = 8.49, *p* = .004, ƞ^2^ = .02). The NoRSA and IndRSA groups did not differ significantly (*M* = 22.15, *SD* = 3.96, *D* = 0.70, *F*(1, 432) = 1.09, *p* = .298, ƞ^2^ < .01).

Similar results were obtained for the memory confidence measured on the VAS scale. The mean memory confidence in the NoRSA group was 57.57 (*SD* = 17.21) and was statistically significantly lower compared to the MemRSA group (*M* = 64.82, *SD* = 20.43, *D* = 7.25, *F*(1, 430) = 7.34, *p* = .007, ƞ^2^ = .02) and the MemIndRSA group (*M* = 66.37, *SD* = 24.08, *D* = 8.80, *F*(1, 430) = 10.68, *p* = .001, ƞ^2^ = .02). There were no statistically significant differences in memory confidence between the NoRSA and IndRSA groups (*M* = 58.74, *SD* = 16.80, *D* = 1.17, *F*(1, 430) = 0.19, *p* = .665, ƞ^2^ < .01). In sum, these results confirm the effectiveness of MemRSA on memory confidence.

The same analyses were performed in order to test the effectiveness of experimental manipulation in inducing a sense of independence. On the VAS scale, the NoRSA group (*M* = 55.16, *SD* = 22.95) scored significantly lower compared to the IndRSA group (*M* = 63.33, *SD* = 20.70, *D* = 8.17, *F*(1, 430) = 7.55, *p* = .006, ƞ^2^ = .02) and the MemIndRSA group (*M* = 65.60, *SD* = 22.80, *D* = 10.44, *F*(1, 430) = 12.34, *p* < .001, ƞ^2^ = .03). The NoRSA group was also found to have a lower subjective independence of judgments compared to the MemRSA group (*M* = 65.31, *SD* = 20.96; *D* = 8.17, *F*(1, 430) = 11.84, *p* = .001, ƞ^2^ = .03).

As regards subjective independence of judgements as measured by the questionnaire, the NoRSA group (*M* = 17.44, *SD* = 3.79) had significantly lower results compared to the IndRSA group (*M* = 21.07, *SD* = 5.69, *D* = 3.63, *F*(1, 430) = 27.50, *p* < .001, ƞ^2^ = .06) as well as in comparison with the MemIndRSA group (*M* = 20.96, *SD* = 6.45, *D* = 3.52, *F*(1, 430) = 25.83, *p* < .001, ƞ^2^ = .06). Interestingly, it was again noted that the NoRSA group had a significantly lower sense of independence compared to MemRSA (*M* = 19.05, *SD* = 4.22, *D* = 1.61, *F*(1, 430) = 5.51, *p* = .019, ƞ^2^ = .01). This may mean that engaging the participant in self-affirmation, combined with positive feedback on the functioning of their memories fosters their sense of independence in the decisions they make. In general, the obtained results indicate the effectiveness of IndRSA in promoting a sense of being an independent person. Contrary to Experiment 1, the four measures relating to manipulation checks did correlate positively (*rs* from .20 to .42, all *ps* < .01).

#### Main analyses

Descriptive results concerning the mean number of answers consistent with misinformation are given in Table 3 (range of the means: 0 to 6).

**Table 3:** Means (*SD*s) of number of answers consistent with misinformation across experimental conditions in Experiment 3 (range: 0–6).

Feedback in RSA	No misinformation	Misinformation	Total
No feedback	0.44 (0.76)	2.15 (1.91)	1.28 (1.67)
Memory	0.45 (0.83)	1.37 (1.40)	0.90 (1.23)
Independence	0.41 (0.69)	1.53 (1.17)	0.96 (1.11)
Memory + Independence	0.31 (0.70)	1.05 (1.47)	0.69 (1.22)
Total	0.40 (0.74)	1.53 (1.56)	0.96 (1.34)

In accordance with Hypothesis 1, the misinformation effect was replicated: the mean number of answers consistent with misinformation was higher in the misled than in the control group (*D* = 1.13, *F*(1, 428) = 96.74, *p* < .001; ƞ^2^ = .18). The second hypothesis was also confirmed, as yielding to misinformation in the misled group was significantly lower in the MemRSA group compared to the NoRSA group: *D* = -0.78, *F*(1, 428) = 11.50, *p* < .001, ƞ^2^ = 0.03.

In order to verify the third hypothesis, the results of the IndRSA and the NoRSA group were compared, and the significant results confirmed the hypothesis (*D* = -0.62, *F*(1, 428) = 7.22; *p* = .007; ƞ^2^ = .02). This replicates the results by Szpitalak and Polczyk [50]. Next, yielding to misinformation was compared between the MemRSA and MemIndRSA groups. The difference was not significant, leaving Hypothesis 4a without support (*D* = -0.32, *F*(1, 428) = 1.91, *p* = .168, ƞ^2^ < .01). To verify Hypothesis 4b, the results of the IndRSA and MemIndRSA groups were compared. The hypothesis was confirmed (*D* = -0.48, *F*(1, 428) = 4.25; *p* = .040; ƞ^2^ = .01). The differences among the RSA groups in the case of non-misled participants were not significant: NoRSA *vs*. MemRSA: *D* = 0.01, *F*(1, 428) < 0.01, *p* = .972, ƞ^2^ < .01; NoRSA *vs*. IndRSA: *D* = -0.03, *F*(1, 428) = 0.02, *p* = .891; ƞ^2^ < .01, NoRSA *vs*. MemIndRSA: *D* = -0.13, *F*(1, 428) = 0.33, *p* = .568, ƞ^2^ < .01; MemRSA *vs*. IndRSA: *D* = -0.04, *F*(1, 428) = 0.03, *p* = .864, ƞ^2^ < .01; MemRSA *vs*. MemIndRSA: *D* = -0.14, *F*(1, 428) = 0.36, *p* = .546, ƞ^2^ < .01; IndRSA *vs*. MemIndRSA: *D* = -0.10, *F*(1, 428) = 0.19, *p* = .667, ƞ^2^ < .01.

Finally, mediation analyses were performed, in a similar manner as in Experiment 1. The predictor was set as a multicategorical variable and indicator contrasts were calculated, with the NoRSA as the reference group, and each of the groups: MemRSA, IndRSA and MemIndRSA groups compared to it. As the mediator variables, aggregated indices of memory confidence and independence of judgements. They were computed as the means of standardized results on the VAS and questionnaires measures. Table 4 presents the results of mediation analyses.

**Table 4:** Results of mediation analysis—Experiment 2.

Contrasts for the predictor	Mediator	*b* [95% CIs]
NoRSA—MemRSA	Memory confidence	-0.36 [-0.56, -0.14]
	Independence of judgements	-0.17 [-0.34, -0.05]
NoRSA—IndRSA	Memory confidence	-0.11 [-0.30, 0.05]
	Independence of judgements	-0.25 [-0.45, -0.08]
NoRSA—MemIndRSA	Memory confidence	-0.36 [-0.60, -0.13]
	Independence of judgements	-0.30 [-0.54, -0.12]

Predictor: RSA; Dependent variable: Number of answers consistent with misinformation

Out of six analyses, five confirmed the hypotheses. MemRSA proved to influence yielding to misinformation *via* memory confidence. The impact of IndRSA on yielding to misinformation was indeed mediated by independence of judgements but not by memory confidence. Finally, both memory confidence and independence of judgements mediated the impact of MemIndRSA on the misinformation effect. The only unexpected, although statistically significant result was that the influence of MemRSA on the misinformation effect was also significantly mediated by independence of judgements.

## General discussion

The misinformation effect consists in including by the witnesses into their testimony false information stemming from other sources that a given event. Research presented in this paper aims at reducing the vulnerability of witnesses to this effect.

First of all, the present research replicated the existence of the misinformation in two experiments. The classical paradigm was applied [6]: the participants watched to a video clip, after some time they were presented with a description of it which in the misled group contained misinformation, and subsequently answered a series of questions about the video, including questions referring to the critical misled items. Participants in the experimental group much more often reported information congruent with the misinformation than did the participants from the control non-misled group. This is yet another warning that the misinformation effect is a robust phenomenon, stable across various alterations and modifications of the experimental procedure and across cultures and quite sizeable.

In two experiments presented in this paper, the reinforced self-affirmation was used to attenuate the misinformation effect. The basic premise for this procedure was the assumption the there are many participants who at the moment of the final memory test remember both the original information as well as the misinformation included in the postevent material (not realizing of course that it is false). As described in Introduction, the existence of participants aware of the discrepancies between the original and postevent materials is now well established [5,27,28]. Also, there are data suggesting that the main reason for giving a wrong answer in this situation is lack of confidence in one’s own memory [5]. The last-mentioned fact was the basic premise for developing a method for decreasing the tendency to give answers consistent with the postevent material, not with one’s own correct memory. This method aims at increasing self-confidence. Increased self-confidence should obviously reduce the tendency to rely on external sources of information if this tendency is caused by low confidence.

The method for increasing self-confidence, which we called reinforced self-affirmation (RSA) consists of two parts: engaging the participants in self-affirmation (by having them write down their greatest achievements in life), and a positive feedback. In the present study, one of the two core elements of RSA, namely positive feedback was analyzed. In the original, most often researched version of RSA, positive feedback concerned memory. This seemed logical, as the aim of RSA was to reduce the vulnerability to misinformation in the context of witness testimony. However, it seemed worth researching whether feedback directed at other cognitive functions, not only memory, would be effective as well. There is at least one reason to research this: self-affirmation in the classical version of RSA was not related to memory. The participants just wrote down their greatest achievements in life, and usually they had nothing to do with memory. Yet, RSA in this form was effective. Existing empirical data suggest that this was not due to the positive feedback directed at memory, because none of the elements of RSA (self-affirmation and positive feedback) was successful when applied alone [41,57].

With this in mind, two new versions of RSA were developed, one with feedback concerning perception abilities, the second—evoking convictions that one is very independent in their judgements, possesses “independent self”. The results of two experiments presented in this paper indicate that RSA with feedback concerning perception was effective and did not significantly differ in efficacy from the classical version with feedback aiming at memory. RSA with feedback promoting a sense of being an independent person was also effective, and again no significant difference in efficacy emerged between feedback concerning memory and independence. However, combining feedbacks concerning memory and independence was more effective that independence alone, although it seemed no more effective than feedback concerning memory alone.

In general, these results indicate that it is not necessary for the feedback to be directed at memory, in order to be effective in immunizing against misinformation in the context of memory reports. It must be stressed however that areas chosen at the present research to be reinforced by positive feedback were not very remote from abilities that may be important for an eyewitness. Obviously, perception abilities are crucial in this context. Also, the ability to rely on one’s own ideas is very important in order not to be suggestible and dependent on external cues while giving a memory report.

Interestingly however, combining inducing confidence about memory and independence of judgements was no more effective than increasing confidence about memory. It may be that boosting self-confidence has its limits. It may also be that it is enough for the witness to reach some level of self-confidence—boosting confidence over and beyond this point does not improve the resistance further.

In sum, the take home message from these results, combined with the results obtained by Szpitalak and Polczyk [52] is that future efforts aiming at developing a version of RSA which could be used in real interrogations should include a positive feedback aiming at cognitive functions and abilities which are not far away from the reality of giving memory reports.

One of the basic premises for the present research was that there are participants who, at the moment of the final memory test for the original material, have a correct memory both for this original material, as well as regards misinformation. The existence of such participants was not directly verified in the experiments presented in this paper. The procedure was quite complex and difficult for the participants. Adding yet another element to it, as required by the four-stage procedure for detecting the awareness of discrepancies, would be too complicated. This is an important point as the main hypothesis posits that RSA actually *only* works in the case of participants aware of discrepancies between the original and postevent materials. Boosting self-confidence may make such participants to rely on their own recollections. If someone does not remember the relevant content of the original material, increased confidence is of little help. In other words, it was assumed that RSA only works on a fraction of participants who are aware of the discrepancies, therefore experience confusions and may start to doubt their own memories. RSA may help such participants to restore confidence that their own memories as regards the original material are correct. This crucial assumption—that RSA only works in the case of participants who are aware of the discrepancies—was not tested in present experiments. There is however data to confirm this basic assumption: Szpitalak and Polczyk [50] were able to show that RSA was only effective in the group of participants aware of the discrepancies between the original and postevent materials.

Another basic premise in the present research was the assumption that its efficacy is based on increasing confidence. This hypothesis was confirmed by the mediation analyses: in most cases they yielded results consistent with this hypothesis: self-confidence and self-independence proved to mediate the impact of RSA on resisting misinformation. In sum, the various pieces of the puzzle seem to fit together: there are data to confirm: (a) the existence of persons aware of the discrepancies between the original and postevent materials [5,27,28]; (b) the fact that RSA is only effective among those aware of the discrepancies [50]; (c) the main reason to reject one’s own memory is lack of confidence [5]; and (d) RSA indeed works *via* increased confidence (present data).

One of the consequences of the fact that RSA is only effective in groups of participants who are aware of the discrepancies between the original and postevent may be that it is actually more powerful than the rather low effect sizes obtained in the present study suggest. If the experiment was performed *only* on aware participants, these effect sizes would most probably be higher. On the other hand, however, this means that RSA would be effective only in the case of a real eyewitness who does remember a given event. This limitation of RSA is difficult to overcome to overcome: it simply is not designed to *increase* memory. It is designed to help witnesses who are confused to help give testimonies based on their own knowledge. Also, RSA does not influence encoding nor storage, as it was administered just before the final memory test. However, there are data suggesting that it is also effective when placed between presenting the original material and exposing the participant to the postevent information [41,57].

The sample sizes used in both experiments are worth a comment. In the first experiment, students of secondary schools took part and their age of the participants ranged from 15 to 18 years (mean age: 16.6). In Experiment 2, students of high schools and other persons were tested, and they were older, ranging in age from 15 to 65 years (mean = 23). RSA was effective in both groups which which suggests that the results concerning RSA generalize across populations.

### Limitations and future directions

The most basic limitation of the present study which immediately calls for future research is the fact that it is basic in its nature. It shows some phenomenon—increasing self-confidence reduces vulnerability to misinformation. However, in the present form it cannot possibly be used in any real situations. It would be very unusual to have a real witness write down their greatest achievements in life, let alone faking them by means of giving manipulated positive feedback. Developing a version of RSA which could be used in real life settings is an important task for future research and the present results may help in it.

RSA consists of two elements, but in the present research, only one of it—positive feedback was systematically researched. Self-affirmation was not manipulated, and it had the same form in both experiments. A better design would have systematically varied the both factors: various kinds of self-affirmation and positive feedback. This however, combined with the misinformation factor which also requires a control group, would result in a too big number of groups. However, it is necessary to study various kinds of self-affirmation as well.

The effect sizes concerning the efficacy of RSA were not large suggesting that the decrease of suggestibility resulting from it is not impressive. However, one can argue that there are few injustices more severe than being wrongly convicted, especially for a serious crime. Trying to save this fate even to several people is worth undertaking.

Another possible limitation of the current study is the fact that it only included one type of false memories—the misinformation effect, that is, errors caused by external misinformation. Whether RSA would be effective in the case of other kinds of false memories (e.g. self-generated ones) remains to be examined.

It would be interesting to examine whether RSA also increases confidence in false memories. This important problem cannot be resolved with the present data as no confidence ratings were collected during the memory test. This remains an important issue for further research.

Another limitation concerning the RSA, affecting first of all the interpretation of the mediation analyses is connected with the method of boosting memory confidence: It consisted of a word-list recall exercise. The participant compared his or her own score to an ostensible low general average. This means that the magnitude of the increase of memory confidence was positively correlated with real memory abilities: participants with poor results on the task were closer to the “average” score than those with good results. As a consequence, the pre-existing memory abilities may have been the real predictor of memory confidence, as well as of yielding to misinformation. The same applies to PerRSA. However, the number of words actually remembered, and the number of differences between pictures correctly solved did not correlate with memory confidence, perception confidence, and yielding to misinformation.

## Supporting information

S1 File Raw data for Experiment 1. (CSV)

S2 File Raw data for Experiment 2. (CSV)

S3 File Descriptions of data files. (TXT)

S4 File Postevent material with misinformation. (PDF)

S5 File Questions on the final memory test. (PDF)
