# Supplementary material for: Inducing resistance to the misinformation effect by means of reinforced self-affirmation: The importance of positive feedback
Source: PLoS One. 2019 Jan 22;14(1):e0210987. doi: 10.1371/journal.pone.0210987 (PMC6342321; doi:10.1371/journal.pone.0210987)
Supplement: S4 File — (PDF) [file pone.0210987.s004.pdf]

The film is about an assault on a jewelry store. First there is a scene in a café. The waitress pretended that she was dropping her eyes, and then she poured some liquid into cups of coffee and gave them to one guy. The guy went out with coffee, passed a street clock at **15:00** (*9:00*) along the way. Then the waitress came out of the cafe, took off the wig, dressed a **green** (*black*) coat and put on sunglasses and a **headkerchief** (*she did not put a headkerchief on*) on her head. Later she looked at the jewelry shop window and read a **book** (*newspaper*) while walking on the pavement. A thick guy threw an explosive into the basket. In a moment it exploded, and then one **red** (*yellow*) car hit another car. There was panic, a woman with a child started to run away, a runaway happened near the broken car, and the guys took advantage of it and started an attack. They opened the door with a crowbar and went into the shop. They started to knock down showcases and shelves and scrap jewelry and **gold watches** (*gold watches were not visible*) into bags. The black man started to drill in the safe. Everything was happening very quickly. The guards were lying unconscious or dead because they probably drank the coffee into which the waitress poured something.

Please note: misleading items are marked in bold and the reality visible in the movie is described in italic in parentheses.
