# Supplementary material for: Inducing resistance to the misinformation effect by means of reinforced self-affirmation: The importance of positive feedback
Source: PLoS One. 2019 Jan 22;14(1):e0210987. doi: 10.1371/journal.pone.0210987 (PMC6342321; doi:10.1371/journal.pone.0210987)
Supplement: S5 File — (PDF) [file pone.0210987.s005.pdf]

1. Did a man or a woman take coffee from the café?

2. With what did the criminals break the door to the shop?

3. With what did the criminals hide the video camera in the shop?

4. What was stolen by the attackers?

5. How many cups of coffee were given to a man in a café?

6. At what time was the attack?

7. What was the colour of the car that hit another one at the moment of the explosion?

8. What did the man who threw the explosives into the dustbin carry?

9. What colour was the waitress's coat?

10. What did the waitress have on her head after leaving the bar and taking off the wig?

11. What did the waitress read while walking down the street?

12. What race were the thieves?

The questions relating to misleading items are marked in bold.
